# The first mitochondrial genome from the Sterrhinae (Lepidoptera: Geometridae) and its phylogenetic implications

**DOI:** 10.1080/23802359.2019.1699467

**Published:** 2019-12-12

**Authors:** Lu Song, Yu-Xia Shi, Jun-Hao Li, Si-Lin Su, Hong-Fei Zhang, Wei-Li Ding, Ming-Sheng Yang

**Affiliations:** College of Life Science and Agronomy, Zhoukou Normal University, Zhoukou, Henan, China

**Keywords:** Geometroidea, mitochondrial genome, moths, phylogeny

## Abstract

In this study, the complete mitochondrial genome of a geometrid species, *Idaea simplicior* (Prout), was sequenced. The *I. simplicior* mitogenome is a circular, double-stranded molecule, with 15,950 bp in size. The typical 37 mitochondrial genes (13 PCGs, 22 tRNAs, and 2 rRNAs) and an A + T-rich region are included. Gene content and arrangement are highly conserved and typical of Lepidoptera. Interestingly, the *I. simplicior* mitogenome is rich in microsatellite sequences of (TA)_12–18_ with a scattered distribution in six intergenic regions. Phylogenetic analyses based on the combined 37 mitochondrial genes show that the Larentiinae and Ennominae are monophyletic. The Sterrhinae, which is represented by the only *I. simplicior* sequenced in this study, shows a closer relationship with the Larentiinae than Ennominae, which confirms previous morphological studies.

The Geometridae, including more than 26,000 described species, is one of the most species-rich groups in Lepidoptera (van Nieukerken et al. 2011; Liu et al. 2014). However, mitochondrial genomes (mitogenomes) representing only 13 species of two geometrid subfamilies were reported. Mitogenome sequences harbor high genetic information (Timmermans et al. 2014) and more taxa with mitogenomes sequenced would effectively contribute to evolutionary studies on this group. In this study, we sequenced the complete mitogenome of an additional geometrid species *Idaea simplicior* (Prout), which represented the first sequenced species of the subfamily Sterrhinae, using next-generation sequencing.

Adult specimens were sampled from Mountain Jigongshan (114°06′56″E, 31°49′25″N) of Henan Province, China. After species identification and the extraction of genomic DNA, one library was constructed, and an Illumina Miseq platform was used for sequencing with the strategy of 250 paired-ends. Voucher specimens are deposited in the Biology Laboratory of Zhoukou Normal University (accession number: 2018JGSA14), China.

The *I. simplicior* mitogenome (GenBank accession number: MN715151) is a circular, double-stranded molecule, with 15,950 bp in size. The typical 37 mitochondrial genes (13 PCGs, 22 tRNAs, and 2 rRNAs) and an A + T-rich region were detected. Gene content and arrangement are highly conserved and typical of Lepidoptera. The nucleotide composition is highly biased toward A/T, with an A + T content of 82%, a feature commonly present in insects (Boore 1999).

The total length of 13 PCGs is 11,203 bp, encoding 3733 amino acids. Most PCGs use the conventional ATN as start codon, with an exception being TTG for the *cox1*. TAA is routinely used as a stop codon, whereas the incomplete termination codon T is recognized in *cox1*, *cox2*, *nad5* and *nad4* genes. Typically, 22 tRNAs are recognized. All tRNAs exhibit typical clover-leaf secondary structure, but *trnS1* (AGN) lacks the DHU arm, which is common in Lepidoptera insects (Garey and Wolstenholme 1989). Two rRNA genes, *rrnS* and *rrnL* with lengths 779 bp and 1406 bp, respectively, were recognized. There are seven overlapping regions ranging from 1 to 8 bp. Interestingly, the *I. simplicior* mitogenome is rich in microsatellite sequences of (TA)_12–18_ with a scattered distribution in six intergenic regions. In particular, the intergenic region (23 bp) between the *trnS2* and *nad1* was also recognized, which is characterized by the presence of the ‘ATACTAA’. The A + T-rich regions of both species contain typical conserved sequence elements such as the motif ‘ATAGA’ and subsequent poly-T structure.

In addition, phylogenetic trees were constructed based on a dataset including all 37 mitochondrial genes of the species sequenced herein together with all other 13 geometrid species, and one epicopeiid species in Geometroidea was selected as an outgroup taxon. Both maximum likelihood and Bayesian inference analyses (Figure 1) consistently recovered that the Larentiinae and Ennominae were monophyletic with high supports. The Sterrhinae, which was represented by the only *I. simplicior* sequenced in this study, showed a closer relationship with the Larentiinae than Ennominae, which confirmed previous morphological studies.

**Figure 1.**
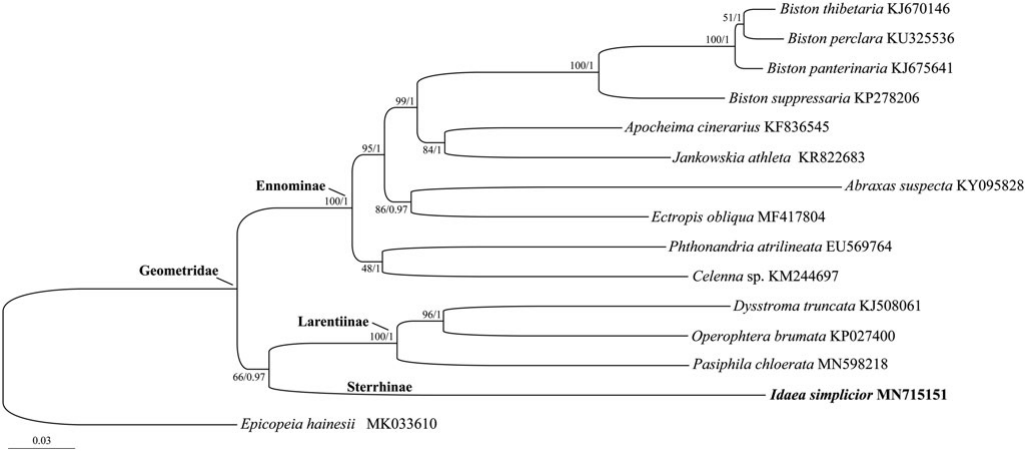
Phylogenetic tree obtained from Bayesian analysis based on the dataset consisting of all 37 mitochondrial genes. The species with newly sequenced mitogenome was emphasized in bold. Numbers separated by a slash on node are bootstrap value for maximum likelihood analysis and posterior probability for Bayesian analysis.
